# Anti-Hepatitis B Virus Effect and Possible Mechanism of Action of 3,4-*O*-Dicaffeoylquinic Acid *In Vitro* and *In Vivo*

**DOI:** 10.1155/2012/356806

**Published:** 2012-06-03

**Authors:** Yi-Hang Wu, Bing-Jie Hao, Hong-Cui Cao, Wei Xu, Yong-Jun Li, Lan-Juan Li

**Affiliations:** ^1^State Key Laboratory for Diagnosis and Treatment of Infectious Disease Key Laboratory of Infectious Diseases, The First Affiliated Hospital, Zhejiang University, School of Medicine, Hangzhou 310003, China; ^2^Department of Pharmacy, College of Life Sciences, China Jiliang University, Hangzhou 310018, China

## Abstract

The anti-hepatitis B activity of 3,4-*O*-dicaffeoylquinic acid isolated from *Laggera alata* was studied using the D-galactosamine- (D-GalN-) induced hepatocyte damage model, HepG2.2.15 cells, and with HBV transgenic mice. *In vitro* results showed that 3,4-*O*-dicaffeoylquinic acid improved HL-7702 hepatocyte viability and markedly inhibited the production of HBsAg and HBeAg. At a concentration of 100 **μ**g/mL, its inhibitory rates on the expression levels of HBsAg and HBeAg were 89.96% and 81.01%, respectively. The content of hepatitis B virus covalently closed circular DNA (HBV cccDNA) in HepG2.2.15 cells was significantly decreased after the cells were treated with the test compound. In addition, 3,4-*O*-dicaffeoylquinic acid significantly increased the expression of heme oxygenase-1 (HO-1) in HepG2.2.15 cells. *In vivo* results indicated that the test compound at concentrations of 100 **μ**g/mL significantly inhibited HBsAg production and increased HO-1 expression in HBV transgenic mice. In conclusion, this study verifies the anti-hepatitis B activity of 3,4-*O*-dicaffeoylquinic acid. The upregulation of HO-1 may contribute to the anti-HBV effect of this compound by reducing the stability of the HBV core protein, which blocks the refill of nuclear HBV cccDNA. Furthermore, the hepatoprotective effect of this compound may be mediated through its antioxidative/anti-inflammatory properties and by the induction of HO-1 expression.

## 1. Introduction

Hepatitis B is an infectious illness caused by hepatitis B virus (HBV), which infects the liver of Hominoidea, including humans, and causes an inflammation reaction called hepatitis. Although there is an effective vaccine against HBV, chronic infection poses a huge health burden on the global community [1]. Its prevalence approaches 10% in hyperendemic areas such as Southeast Asia, China, and Africa [2]. Furthermore, approximately one-third of the world's population (more than 2 billion people) have been infected with the hepatitis B virus, which includes 350 million chronic carriers of the virus [3]. Some antiviral agents such as interferon-*α* and nucleosides (including lamivudine and adefovir dipivoxil) have been approved for the treatment of chronic HBV infection. However, a significant number of patients develop drug resistance after long-term use of these agents [4]. Therefore, there is a pressing need to continue developing safer and more effective anti-hepatitis B agents. The development of natural substances as antiviral agents is thought to be a promising approach towards solving this public health concern [5].

**Figure 1:**
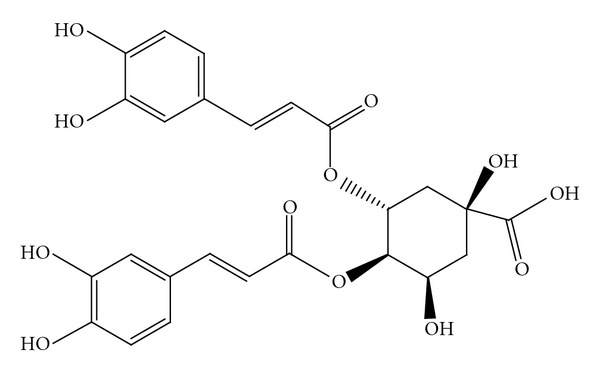
Structure of 3,4-*O*-dicaffeoylquinic acid isolated from *L. alata*.

*Laggera alata *belongs to the genus *Laggera* (Asteraceae) and is distributed mainly among the tropical areas of Africa, Southeast Asia, and China. This plant has been used as a folk medicine for over 300 years, especially for the treatment of some ailments associated with hepatitis [6]. Most of the previous studies examining *L. alata* have focused on its folk use and phytochemical analyses [7–9]. In previous investigations, we examined the anti-inflammatory and hepatoprotective activities of an *L. alata* extract containing dicaffeoylquinic acids and confirmed its potent effects [10, 11]. In this study, we utilized the d-galactosamine- (d-GalN-) induced HL-7702 hepatocyte damage model, HBV-transfected HepG2.2.15 cells, and HBV transgenic mice to evaluate the anti-hepatitis B activity and possible hepatoprotective mechanisms of 3,4-*O*-dicaffeoylquinic acid isolated from* L. alata *(Figure 1). The study is the first to demonstrate that 3,4-*O*-dicaffeoylquinic acid possesses an anti-hepatitis B activity.

## 2. Materials and Methods

### 2.1. Reagents

Fetal bovine serum, Dulbecco's modified Eagle's medium (DMEM) and 1640 medium were purchased from Gibco-BRL (Grand Island, NY, USA). 3-(4,5-Dimethylthiazol-2-yl)-2,5-diphenyltetrazolium bromide (MTT), silibinin, and d-galactosamine were purchased from Sigma Chemical Co., USA. Lamivudine was provided by GlaxoSmithKline Investment Co., Ltd. The HBV DNA PCR-fluorescence quantitation kit and the enzyme immunoassay (EIA) kits for the detection of HBsAg, and HBeAg were obtained from Shanghai Kehua Bio-Engineering Co., Ltd. The Plasmid Mini Preparation kit was obtained from Axygen Biosciences. Plasmid safe ATP-dependent DNase was purchased from EPICENTRE Biotechnologies. The TA cloning kit was obtained from Invitrogen Corporation. The heme oxygenase-1 ELISA kit was purchased from Beijing Yonghui Biological Technology Co., Ltd. Conventional PCR reagents were obtained from Shanghai Sangon Biological Engineering Technology and Service Co., Ltd. The HBsAg immunohistochemical detection kit was purchased from Boster Biological Technology Co., Ltd., China. All other reagents were of the highest available commercial grade.

### 2.2. Compound

3,4-*O*-Dicaffeoylquinic acid was isolated from *Laggera alata* and its structure was authenticated according to a previously reported method [10]. All of the *Laggera alata* (D. Don) Sch.-Bip ex Olivier was collected from Yunnan Province, China. A voucher specimen (ZY982003LA) was deposited in the herbarium of the College of Pharmaceutical Sciences, Zhejiang University, China. 3,4-*O*-Dicaffeoylquinic acid (HPLC purity ≥ 98%) was initially dissolved in dimethyl sulfoxide (DMSO) and further diluted in cell culture medium to achieve a final concentration of 0.1% DMSO, which was not toxic to either HL-7702 hepatocytes or HepG2.2.15 cells.

### 2.3. Cells and Transgenic Mice

HL-7702 hepatocytes were maintained in 1640 medium containing 2 mM glutamine and 10% (v/v) fetal bovine serum at 37°C (95% humidity, 5% CO_2_). HepG2.2.15 cells were maintained in DMEM containing 2 mM glutamine, 10% (v/v) fetal bovine serum, and 380 *μ*g/mL of G418 at 37°C (95% humidity, 5% CO_2_). HBV transgenic mice were generated in the Shanghai Research Center for Model Organisms by routine microinjection of the linearized HBV DNA of clone no. 25-8 (GenBank ID: AF461363) into fertilized eggs of C57BL/6J mice [12]. The transgenic mice were kept in a room maintained at 22 ± 2°C and at relative humidity between 40% and 70%. The experimental protocol was approved by the Animal Ethics Committee of Zhejiang University, in accordance with “Principles of Laboratory Animal Care and Use in Research” (Ministry of Health, Beijing, China).

### 2.4. Hepatoprotective Assay against d-GalN-Induced Hepatocyte Damage

HL-7702 hepatocytes were transferred to 96-well plates at a density of approximately 1.0 × 10^5^ cells/mL. Cytotoxicity induced by the test compound was measured using the MTT assay as reported previously [11]. Hepatocyte injury was induced by d-GalN in the following manner: after HL-7702 hepatocytes were incubated for 8 h with 80 mM d-GalN, the cells were then cultured for another 48 h in fresh culture medium containing 1–100 *μ*g/mL 3,4-*O*-dicaffeoylquinic acid. Hepatocyte viability was detected using the MTT assay. The hepatoprotective effect of the test compound was assessed by the cell viability assay and expressed as percent protection. Silibinin was used as the reference drug at a concentration of 100 *μ*g/mL.

### 2.5. Anti-HBV Assay in HepG2.2.15 Cells

 Cytotoxicity induced by 3,4-*O*-dicaffeoylquinic acid was analyzed as follows: HepG2.2.15 cells were transferred to 96-well plates at a concentration of 1.0 × 10^5^ cells/mL. Different concentrations of the test compound were applied to the culture wells in triplicate. After the cells were incubated for 8 days, the MTT assay was carried out as described previously [11]. To measure the effect of 3,4-*O*-dicaffeoylquinic acid on the expression of HBV antigens and HBV DNA, HepG2.2.15 cells were treated with various concentrations of the test compound in the 96-well plates. The medium with the compound was replaced every 4 days. On the fourth day, the replaced medium was assayed for HBsAg and HBeAg. On the eighth day, the replaced medium was measured for HBsAg, HBeAg and HBV DNA. Lamivudine was used as the reference drug. The levels of HBsAg and HBeAg in the replaced culture supernatants were determined by HBsAg and HBeAg enzyme-immunoassay kits, respectively. The results were read at 450 nm by a multiwell plate reader (MULTISKAN MK3, Thermo Fisher Scientific Inc., USA). The HBV viral load in the replaced culture supernatants was detected with a HBV DNA PCR-fluorescence quantitation kit as follows: HBV DNA was extracted and amplified with a Bio-Rad iQ5 real-time PCR system. The forward primer was 5-CCG TCT GTG CCT TCT CAT CTG-3, the reverse primer was 5-AGT CCA AGA GTA CTC TTA TAG AAG ACC TT-3, and the Taqman probe was FAM-CCG TGT GCA CTT CGC TTC ACC TCT GC. The thermal program comprised of an initial denaturation at 94°C for 2 min followed by 40 amplification cycles with each of the two following steps: 95°C for 5 s and 60°C for 30 s. A plasmid containing the full-length insert of the HBV genome was used to prepare the standard curve.

### 2.6. Assay for Elimination HBV cccDNA in HepG2.2.15 Cells

After HepG2.2.15 cells were incubated for 48 h in 6-well plates at a density of 1.0 × 10^5^ cells/mL, new DMEM medium containing different concentrations of the test compound (50, 25, and 10 *μ*g/mL) was added. Three parallel controls were performed, including positive controls with oxymatrine (50 *μ*g/mL), a vehicle control of 0.1% DMSO, and a normal control with no antiviral drug. The medium with the compound was replaced every 3 days. On the sixth day, the cells of each well were harvested. Based on the similarity of cccDNA and plasmid structures, the cell pellet containing 1.0 × 10^6^ cells was extracted with the Mini Plasmid Extraction Kit. The extracted plasmid was further purified by plasmid safe ATP-dependent DNase to remove the residual HBV relaxed circular DNA. Hepatitis B virus covalently closed circular DNA (HBV cccDNA) was detected by selective real-time fluorescent quantitative PCR with specific primers and a Taqman MGB probe [13]. The forward primer was 5-TGA ATC CTG CGG ACG ACC-3, the reverse primer was 5-ACA GCT TGG AGG CTT GAA CAG-3 and the Taqman probe was 5-FAM-CCT AAT CAT CTC TTG TTC ATG TC-MGB-3. According to the structural differences between cccDNA and rcDNA, only the cccDNA should have been amplified with the designed primers and probe.

### 2.7. Assay for Induction of HO-1 of HepG2.2.15 Cell

After HepG2.2.15 cells were incubated for 48 h in 6-well plates at a density of 1.0 × 10^5^ cells/mL, the cells were treated with various concentrations of test compound, and the medium with the compound was replaced every 3 days. Oxymatrine was used as the reference drug. On the sixth day, the cells were collected, and their heme oxygenase-1 (HO-1) levels were determined by an HO-1 ELISA kit according to the protocol provided with the kit. The absorbency was measured at 450 nm by a multiwell plate reader. The content of HO-1 in these cells was then determined by comparing the absorbency of the samples to the standard curve.

### 2.8. Anti-HBV Assay in HBV Transgenic Mice

 HBV transgenic mice were divided into four groups. The vehicle control group received a normal saline solution at a dose of 10 mL/kg. The drug control group received lamivudine at a dose of 100 mg/kg. Experimental drug groups received 3,4-*O*-dicaffeoylquinic acid at doses of 50 and 100 mg/kg. The vehicle and drugs were administered orally to the different groups of mice once per day for 30 days. Five hours after the last administration, the mice were briefly anesthetized with ether, and blood samples were taken from the orbital sinus. The serum was separated for the measurements of HBsAg, and HO-1. The serum HBsAg and HO-1 levels were determined using the HBsAg and HO-1 detection kits according to the respective protocols provided with the ELISA kits. For histopathological analysis, formalin-fixed, paraffin-embedded liver specimens were routinely stained with hematoxylin and eosin (HE). The liver HBsAg expression level was determined using the HBsAg immunohistochemical detection kit according to the manufacturer's instructions. The pathological and immunohistochemical changes were evaluated and photographed under the microscope.

### 2.9. Statistical Analysis

 Data were expressed as the mean ± standard deviation. Statistical analyses were carried out by the application of one-way analysis of variance (ANOVA) and student's *t*-test. *P* < 0.05 was chosen as the criterion for statistical significance.

## 3. Results

### 3.1. Effect of 3,4-*O*-Dicaffeoylquinic Acid on d-GalN-Induced Hepatocyte Damage

 The cytotoxicity test indicated that 3,4-*O*-dicaffeoylquinic acid was not toxic to HL-7702 hepatocytes at concentrations of 10–100 *μ*g/mL (Table 1). Hepatocyte injury was induced by exposure to 80 mM d-GalN, and the cells were subsequently treated with 3,4-*O*-dicaffeoylquinic acid. The results show that test compound improved cell viability at concentrations of 10–100 *μ*g/mL (Table 2).

### 3.2. Anti-HBV Activity of 3,4-*O*-Dicaffeoylquinic Acid in HepG2.2.15 Cells

 After hepG2.2.15 cells were treated with 3,4-*O*-dicaffeoylquinic acid for 8 days, the cell viability was determined using the MTT assay. The results indicated that 3,4-*O*-dicaffeoylquinic acid was not cytotoxic at concentrations of 10–100 *μ*g/mL (Table 3). The HBsAg and HBeAg levels in culture supernatants were assayed after the cells were incubated with the test compound for 4 days (Table 4). The results showed that the test compound significantly inhibited HBsAg expression at concentrations of 50–100 *μ*g/mL and markedly repressed HBeAg expression at a concentration of 100 *μ*g/mL.

The HBsAg, HBeAg and HBV DNA levels in culture supernatants were measured after the cells were treated with the test compound for 8 days (Table 5). At concentrations of 50–100 *μ*g/mL, the test compound significantly inhibited the expression of HBsAg and HBeAg. At a concentration of 100 *μ*g/mL, the test compound inhibited the expression rates of HBsAg and HBeAg by 89.96% and 81.01%, respectively.

### 3.3. Effect of 3,4-*O*-Dicaffeoylquinic Acid on HBV cccDNA Content of HepG2.2.15 Cells

 The effect of 3,4-*O*-dicaffeoylquinic acid on the level of HBV cccDNA is shown in Table 6. The results indicated that 3,4-*O*-dicaffeoylquinic acid significantly reduced the HBV cccDNA content of HepG2.2.15 cells at a concentration of 50 *μ*g/mL. Furthermore, the test compound exhibited a larger effect than the reference drug oxymatrine.

### 3.4. Effect of 3,4-*O*-Dicaffeoylquinic Acid on HO-1 Expression in HepG2.2.15 Cell

The expression level of HO-1 in HepG2.2.15 cells was determined after the cells were treated with various concentrations of test compound for 6 days (Table 7). At concentrations of 10–50 *μ*g/mL, 3,4-*O*-dicaffeoylquinic acid significantly increased HO-1 expression. Oxymatrine, which was the reference drug, showed a similar effect.

### 3.5. Anti-HBV Activity of 3,4-*O*-Dicaffeoylquinic Acid in HBV Transgenic Mice

**Figure 2:**
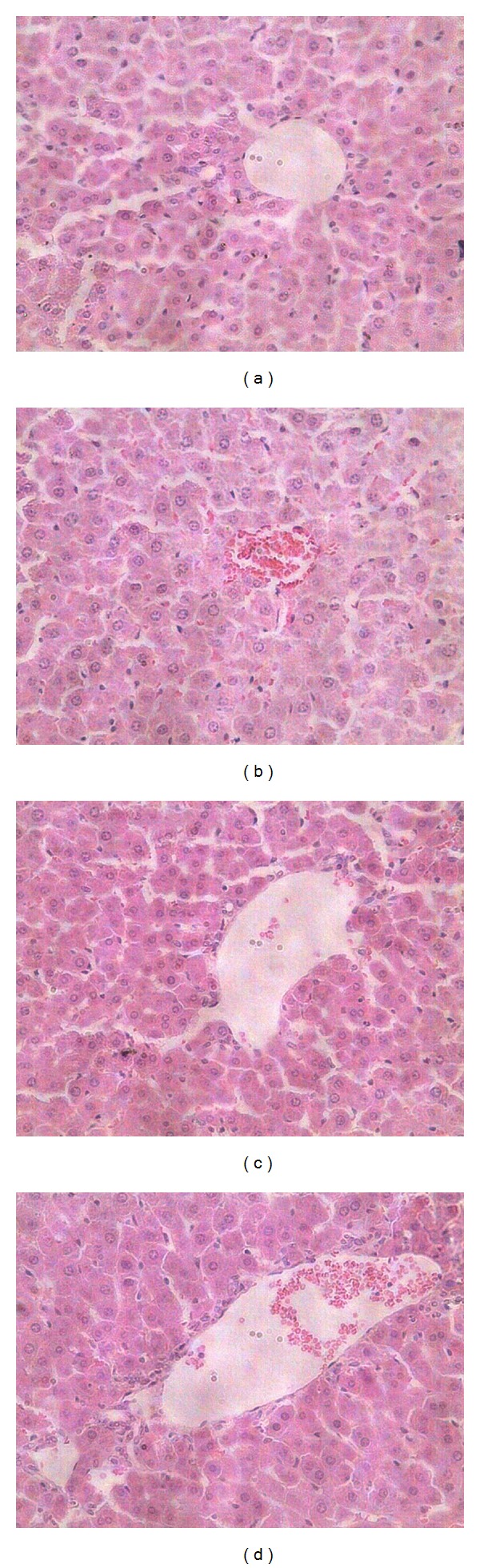
Histopathological changes of liver tissue from HBV transgenic mice (HE × 40). (a) A control untreated HBV transgenic mouse; (b) a lamivudine (100 mg/kg) treated HBV transgenic mouse; (c) a 3,4-dicaffeoylquinic acid (100 mg/kg) treated HBV transgenic mouse; (d) a 3,4-dicaffeoylquinic acid (50 mg/kg) treated HBV transgenic mouse. (a), (b), (c), and (d) do not show obvious pathological changes, which is probably related to the immunotolerance of HBV transgenic mice to HBV.

**Figure 3:**
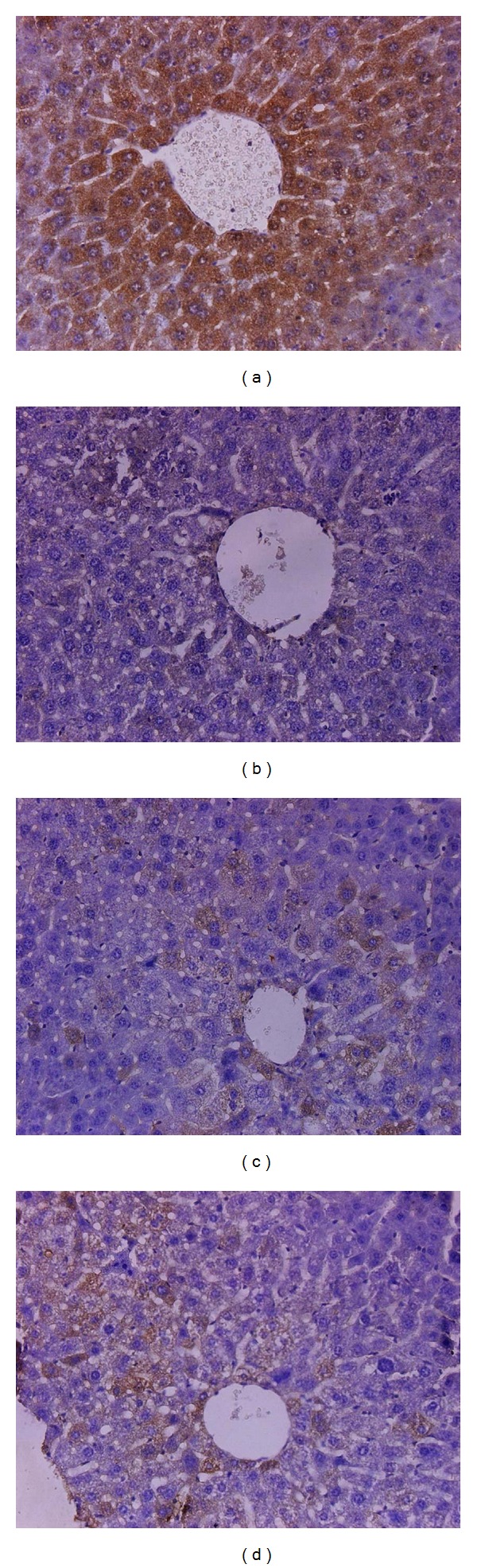
Immunohistochemical staining of HBsAg in the liver of HBV transgenic mice (×40). (a) A control untreated HBV transgenic mouse showing the positive expression of HBsAg (brown stain); (b) a lamivudine (100 mg/kg) treated HBV transgenic mouse; (c) a 3,4-dicaffeoylquinic acid (100 mg/kg) treated HBV transgenic mouse; (d) a 3,4-dicaffeoylquinic acid (50 mg/kg) treated HBV transgenic mouse. (b), (c), and (d) show clear inhibition of HBsAg expression.

 The anti-HBV activity of 3,4-*O*-dicaffeoylquinic acid was determined in HBV transgenic mice (Table 8). These results show that the test compound significantly reduced the serum HBsAg level at concentrations of 50–100 *μ*g/mL. Meanwhile, the test compound markedly induced HO-1 expression at a concentration of 100 *μ*g/mL. Histological analysis revealed almost normal lobule architecture and slight swelling of liver cells, but no obvious pathological changes were observed in the control and drug-treated mice (Figure 2). Immunohistochemical detection indicated that the strongest HBsAg-positive signals were detected in the control group, and the different concentrations of test compound clearly repressed the expression of liver HBsAg (Figure 3).

## 4. Discussion

Patients with hepatitis B are often treated with antiviral agents, hepatoprotective drugs, and immunomodulatory drugs. Typically, the beneficial role of hepatoprotectors in viral hepatitis is achieved through their inhibitory action on the inflammatory and cytotoxic cascades induced by viral infection. In addition, these agents can also improve the regeneration process and normalize liver enzymes through their effects on protein synthesis [14]. Among the numerous models of experimental hepatitis, d-GalN-induced liver damage is very similar to human viral hepatitis in its morphological and functional features [15]. d-GalN reduces the intracellular pool of uracil nucleotides in hepatocytes, thereby inhibiting the synthesis of RNA and proteins [16]. Oxygen-derived free radicals released from activated hepatic macrophages are the primary cause of d-GalN-induced liver damage [17]. In previous studies, the potent anti-inflammatory and hepatoprotective activities of an *L. alata* extract containing dicaffeoylquinic acids were confirmed [10, 11]. Furthermore, dicaffeoylquinic acids exhibit a variety of pharmacological activities, such as antioxidative, anti-inflammatory, and antiviral effects [18–20]. In the current study, 3,4-*O*-dicaffeoylquinic acid protected d-GalN-injured hepatocytes, thereby implying that its antioxidative and anti-inflammatory properties may have contributed to the amelioration of hepatocyte damage.

HepG2.2.15 cells are derived from human hepatoblastoma HepG2 cells that were transfected with a plasmid containing HBV DNA. These cells can stably secrete viral particles in culture medium [21]. The presence of HBsAg is the most common marker of HBV infection, whereas HBeAg is used as an ancillary marker primarily to indicate active HBV replication and associated progressive liver disease [22]. Hepadnaviruses have a relaxed circular DNA genome. Following infection of hepatocytes, this DNA is transported to the nucleus and converted to a covalently closed form (cccDNA) that serves as a transcriptional template. Viral DNA is synthesized within nucleocapsids via reverse transcription of a viral RNA known as the pregenome [23]. Nucleocapsids containing mature forms of viral DNA are packaged into viral envelopes and secreted from the cell. cccDNA does not replicate; however, additional copies (up to 50 per cell) may be formed from viral DNA synthesized in the cytoplasm [24]. The cccDNA plays a key role in the life cycle of the virus and permits the persistence of infection. The formation of cccDNA is inhibited by viral envelope proteins [25]. In this study, 3,4-*O*-dicaffeoylquinic acid significantly inhibited expression of HBsAg and HBeAg in HepG2.2.15 cells. Although the response of lamivudine on HBV DNA replication is on the low side, its suppression on HBV DNA replication is statistically significant when compared to the vehicle group. Under the different experimental conditions, the test results of the same compound may have certain difference because of the different cell states. The low response of HBV DNA to lamivudine does not affect the judgement of the results, only if the anti-HBV activities of lamivudine and test compound are measured and compared under the same test condition. Furthermore, the test compound also significantly reduced the HBV cccDNA content of HepG2.2.15 cells and its effect was stronger than the reference drug oxymatrine, thereby indicating the anti-HBV effect of this compound was probably related to inhibiting the formation of cccDNA.

HBV transgenic mice with a known genetic background and a well-characterized HBV isolate have been employed as an animal model of the HBV-carrier state and are thought to be a good model to evaluate the anti-HBV efficacy of candidate compounds *in vivo *[12, 26]. Based on *in vitro* results, we further studied the anti-hepatitis B activity of 3,4-*O*-dicaffeoylquinic acid in HBV transgenic mice. The results indicated that 3,4-*O*-dicaffeoylquinic acid significantly inhibited the serum and liver HBsAg levels and significantly increased HO-1 expression in these transgenic mice, which was in good agreement with the results of *in vitro* research. Upon histopathological analysis, no obvious pathological changes were found in both the control and experimental groups of mice, which is probably related to the immunotolerance of HBV transgenic mice to HBV. Because these transgenic mice are immunotolerant to HBV, they did not present with any of the disease signs that would normally be associated with immunopathological responses [27].

Heme oxygenases catalyze the initial and rate-limiting step in the oxidative degradation of heme. Among the three known heme oxygenases, HO-1 is the only inducible form of these enzymes [28]. Overexpression of HO-1 protects organs and/or tissues from immune-mediated organ injury, which can occur either through the prevention of oxidative damage or *via* local immunomodulatory influence on inflammatory cells [29]. Induction of HO-1 has been shown to be beneficial in immune-mediated liver damage. In addition, liver injury was significantly reduced after HO-1 induction in an acute hepatitis B model [30]. In addition to its hepatoprotective effect, HO-1 exhibited a pronounced antiviral effect, which was confirmed in stably HBV-transfected hepatoma cells and in persistently HBV replicating transgenic mice. HO-1 induction repressed HBV replication directly in hepatocytes at a posttranscriptional step by reducing the stability of the HBV core protein, which resulted in blocking the refill of nuclear HBV cccDNA. Small interfering RNAs directed against HO-1 have demonstrated that this effect is dependent on the expression level of HO-1 [30]. Therefore, the induction of HO-1 might be a novel therapeutic option for inflammatory flares of hepatitis B. In this study, 3,4-*O*-dicaffeoylquinic acid significantly increased the expression of HO-1 *in vitro *and *in vivo*, thereby suggesting that the hepatoprotective and anti-HBV effects of 3,4-*O*-dicaffeoylquinic acid were achieved by HO-1 induction.

In conclusion, this study verifies the* in vitro* and *in vivo* anti-hepatitis B effects of 3,4-*O*-dicaffeoylquinic acid isolated from *L. alata*. The upregulation of HO-1 may contribute to the anti-HBV effect of this compound by reducing the stability of the HBV core protein and by blocking the refill of nuclear HBV cccDNA. Additionally, the hepatoprotective effect of this compound was mediated by its antioxidative/anti-inflammatory properties and the induction of HO-1. Therefore, 3,4-*O*-dicaffeoylquinic acid should be considered a potential candidate or lead compound for the development of novel antiviral agents.

## Figures and Tables

**Table 1:** Cytotoxicity of 3,4-dicaffeoylquinic acid in HL-7702 hepatocytes.

Groups	Concentration (*μ*g/mL)	Absorbency (570 nm)	Cell survival (%)
Vehicle	—	1.275 ± 0.042	100
Silybin	100	1.235 ± 0.069	96.86
	50	1.238 ± 0.038	97.10
	10	1.259 ± 0.048	98.74
3,4-Dicaffeoylquinic acid	100	1.230 ± 0.048	96.47
	50	1.246 ± 0.027	97.72
	10	1.255 ± 0.046	98.43

All determinations were performed in six replicates, and values were expressed as mean ± SD. No significant difference compared with the vehicle control.

**Table 2:** Effect of 3,4-dicaffeoylquinic acid on the survival of d-GalN injured HL-7702 hepatocytes.

Groups	Concentration (*μ*g/mL)	Absorbency (570 nm)	Protection rate (%)
Vehicle	—	1.289 ± 0.055**	—
d-GalN-control	—	0.769 ± 0.053	—
Silybin-d-GalN	100	0.868 ± 0.036*	18.90
	50	0.832 ± 0.055	12.07
	10	0.810 ± 0.031	7.79
3,4-Dicaffeoylquinic acid- d-GalN	100	0.846 ± 0.044	14.77
	50	0.812 ± 0.031	8.18
	10	0.801 ± 0.019	6.16

Silybin was used as the positive control. 0.1% DMSO was used as the vehicle control. Values are expressed as the means ± SD of four replicates. Protection rate (%) = (the mean absorbency value in experimental group − the mean absorbency value in model control group)/(the mean absorbency value in negative control group − the mean absorbency value in model control group) × 100%. *P < 0.05 and **P < 0.01 represent the significance of the difference from the d-GalN control.

**Table 3:** Cytotoxicity of 3,4-dicaffeoylquinic acid in hepG2.2.15 cells.

Groups	Concentration (*μ*g/mL)	Absorbency (570 nm)	Cell survival (%)
Vehicle	—	0.922 ± 0.031	100
Lamivudine	100	0.906 ± 0.101	98.26
	50	0.912 ± 0.092	98.92
	10	0.918 ± 0.141	99.57
3,4-Dicaffeoylquinic acid	100	0.893 ± 0.061	96.85
	50	0.895 ± 0.105	97.07
	10	0.909 ± 0.078	98.59

All determinations were performed in six replicates, and values were expressed as mean ± SD. No significant difference compared with the vehicle control.

**Table 4:** Anti-HBV activity of 3,4-*O*-dicaffeoylquinic acid in HepG2.2.15 cells. (After the cells were treated with the test compound for 4 days.)

Groups	Concentration (*μ*g/mL)	HBsAg		HBeAg	
		Absorbency	Inhibition (%)	Absorbency	Inhibition (%)
Vehicle	—	1.175 ± 0.085	—	2.947 ± 0.273	—
Lamivudine	100	1.058 ± 0.101	10.01	3.132 ± 0.034	—
	50	1.097 ± 0.083	6.67	3.140 ± 0.039	—
	10	1.197 ± 0.116	—	3.274 ± 0.100	—
3,4-Dicaffeoylquinic acid	100	0.585 ± 0.024**	50.23	2.070 ± 0.198*	29.77
	50	0.840 ± 0.020**	28.50	2.505 ± 0.077	15.02
	10	1.111 ± 0.096	5.48	2.750 ± 0.250	6.70

Lamivudine was used as the positive control in the anti-HBV assay. 0.1% DMSO was used as the vehicle control. Inhibition (%) = (the mean absorbency value in negative control group − the mean absorbency value in experimental group)/(the mean absorbency value in negative control group) × 100%. Data are expressed as the means ± SD of three independent experiments. *P < 0.05 and **P < 0.01 compared with the vehicle group.

**Table 5:** Anti-HBV activity of 3,4-*O*-dicaffeoylquinic acid in HepG2.2.15 cells. (After the cells were treated with the test compound for 8 days.)

Groups	Concentration (*μ*g/mL)	CC_50 _	HBsAg				HBeAg				cccDNA (Log) (copy/*μ*L)
			Absorbency	Inhibition (%)	IC_50_	SI	Absorbency	Inhibition (%)	IC_50_	SI	
Vehicle	—	—	1.023 ± 0.062	—	—	—	3.213 ± 0.109	—	—	—	5.10 ± 0.04

Lamivudine	100	>400	0.867 ± 0.008*	15.19	—	—	3.211 ± 0.195	—	—	—	4.57 ± 0.02**
	50		1.045 ± 0.060	—			3.388 ± 0.082	—			4.65 ± 0.04**
	10		1.078 ± 0.157	—			3.510 ± 0.054	—			4.77 ± 0.11*

3,4-Dicaffeoylquinic acid	100	>400	0.103 ± 0.010**	89.96	31.90	>12.54	0.610 ± 0.060**	81.01	50.06	>7.99	5.04 ± 0.05
	50		0.342 ± 0.026**	66.56			2.081 ± 0.095**	35.21			5.11 ± 0.04
	10		0.910 ± 0.078	11.02			2.894 ± 0.177	9.92			5.14 ± 0.14

Lamivudine was used as the positive control in anti-HBV assay. 0.1% DMSO was used as the vehicle control. CC_50 _(*μ*g/mL): value of the 50% cytotoxic concentration. Inhibition (%) = (the mean absorbency value in negative control group − the mean absorbency value in experimental group)/(the mean absorbency value in negative control group) × 100%. IC_50_ (*μ*g/mL): value of the 50% inhibition concentration. SI: selectivity index (CC_50_/IC_50_). Data are expressed as means ± SD of three independent experiments. *P < 0.05 and **P < 0.01 compared with the vehicle group.

**Table 6:** Effect of 3,4-dicaffeoylquinic acid on the HBV cccDNA content of HepG2.2.15 cells.

Groups	Concentration (*μ*g/mL)	HBV cccDNA (Log) (copy/*μ*L)
Normal	—	2.79 ± 0.03
Vehicle	—	2.73 ± 0.02
Oxymatrine	50	2.59 ± 0.12*
3,4-Dicaffeoylquinic acid	50	2.54 ± 0.05**
	25	2.71 ± 0.08
	10	2.83 ± 0.03

Oxymatrine was used as the positive control. 0.1% DMSO was used as the vehicle control. Data are expressed as the means ± SD of three independent experiments. *P < 0.05 and **P < 0.01 compared with the vehicle group.

**Table 7:** Effect of 3,4-dicaffeoylquinic acid on HO-1 expression in HepG2.2.15 cells. (After the cells were treated with the test compound for 6 days.)

Groups	Concentration (*μ*g/mL)	HO-1 content (ng/g protein)
Normal	—	36.00 ± 0.45
Vehicle	—	33.61 ± 1.51
Oxymatrine	50	57.70 ± 3.21**
	25	51.09 ± 4.80**
	10	49.49 ± 1.99**
3,4-Dicaffeoylquinic acid	50	57.18 ± 3.37**
	25	48.31 ± 2.52**
	10	45.12 ± 0.87**

Oxymatrine was used as the positive control. 0.1% DMSO was used as the vehicle control. Data are expressed as the means ± SD of three independent experiments. *P < 0.05 and **P < 0.01 compared with the vehicle group.

**Table 8:** Anti-HBV activity of 3,4-*O*-dicaffeoylquinic acid in HBV transgenic mice.

Groups	Concentration (mg/kg)	HBsAg	HO-1 (ng/L)
		P/N	
Vehicle	—	55.33 ± 1.10	609.62 ± 39.54
Lamivudine	100	56.82 ± 1.67	646.53 ± 29.19
3,4-Dicaffeoylquinic acid	100	53.16 ± 1.15**	676.31 ± 31.81*
	50	53.62 ± 1.49*	630.51 ± 56.48

Lamivudine was used as the positive control in the anti-HBV assay. Normal saline solution was used as the vehicle control. P/N (positive-to-negative) ratios were determined as the mean absorbency value of the test compounds divided by that of the negative control. Data are expressed as the means ± SD of four samples. *P < 0.05 and **P < 0.01 compared with the vehicle group.
